# Circulating Myonectin and Oxytocin Levels in Pediatric Obesity: A Comparative Study

**DOI:** 10.3390/children13030401

**Published:** 2026-03-13

**Authors:** Muammer Buyukinan, Ummugulsum Can, Zafer Bagci, Sadinaz Akdu

**Affiliations:** 1 Department of Pediatric Endocrinology, Medical Faculty, Selcuk University, Konya 42130, Turkey; 2 Department of Biochemistry, University of Health Sciences, Konya City Hospital, Konya 42020, Turkey; ummugulsum.can1@saglik.gov.tr; 3 Department of Pediatrics, University of Health Sciences, Konya City Hospital, Konya 42020, Turkey; zafer.bagci@sbu.edu.tr; 4 Department of Biochemistry, Fethiye State Hospital, Mugla 48300, Turkey; sadinazakdu@fethiyedh.gov.tr

**Keywords:** pediatric obesity, myonectin, oxytocin, insulin resistance

## Abstract

**Highlights:**

**What are the main findings?**

• Children with obesity exhibited significantly lower circulating myonectin levels compared to healthy controls.
• Circulating oxytocin levels were significantly higher in obese children and were independent of BMI-SDS and HOMA-IR.

**What are the implications of the main findings?**

• Pediatric obesity may involve early alterations in muscle-derived metabolic signaling and neuroendocrine regulation beyond adipose tissue expansion.
• Myonectin and oxytocin may serve as complementary biomarkers reflecting metabolic adaptation in childhood obesity.

**Abstract:**

**Background/Objectives**: The development of obesity is not only related to excessive adipose tissue accumulation but also involves complex inter-organ signaling pathways linking skeletal muscle and neuroendocrine systems. The present study aimed to evaluate circulating levels of myonectin (CTRP15), a skeletal muscle–derived metabolic regulator, and oxytocin, a neuropeptide with anorexigenic properties, in children with obesity. In addition, we examined the potential associations of these biomarkers with insulin resistance and metabolic risk indicators. **Methods**: This cross-sectional study included 53 children with obesity (body mass index standard deviation score [BMI-SDS] > 2) and 37 healthy children with normal body weight serving as controls. Anthropometric parameters, fasting glucose, insulin, lipid profile, and the Homeostasis Model Assessment of Insulin Resistance (HOMA-IR) index were assessed in all participants. Circulating concentrations of myonectin and oxytocin were measured and compared between groups, and correlations with metabolic variables were explored. **Results**: Children with obesity exhibited a less favorable metabolic profile characterized by higher HOMA-IR values, hyperinsulinemia, and elevated triglyceride levels. Serum myonectin concentrations were significantly lower in the obesity group compared with controls (4.01 ± 3.66 vs. 8.35 ± 12.00 ng/mL; *p* = 0.019). In contrast, circulating oxytocin levels were significantly higher among children with obesity (median [IQR] 156.2 [83.9–754.9] vs. 141.7 [47.7–221.5] pg/mL; *p* = 0.044). Neither hormone demonstrated a significant linear relationship with age, BMI-SDS, or HOMA-IR. **Conclusions**: Our findings indicate that childhood obesity is associated with reduced circulating myonectin levels and increased oxytocin concentrations. These observations suggest potential alterations in both muscle-derived metabolic signaling and neuroendocrine regulation in pediatric obesity. However, due to the cross-sectional design of the present study, causal relationships cannot be established.

## 1. Introduction

Childhood obesity represents a major global health concern and is associated with the early development of cardiometabolic complications, including insulin resistance, dyslipidemia, type 2 diabetes, and metabolic dysfunction–associated steatotic liver disease (MASLD) [1,2]. Although obesity has traditionally been explained by positive energy balance and excess adipose tissue accumulation, increasing evidence suggests that its pathophysiology involves complex endocrine interactions among multiple organs. In particular, communication between skeletal muscle, adipose tissue, and the central nervous system has emerged as an important regulatory network influencing metabolic homeostasis [3,4]. Skeletal muscle accounts for a large proportion of total body mass and is increasingly recognized as an active endocrine organ capable of influencing systemic metabolism. Through the secretion of signaling molecules known as myokines, skeletal muscle contributes to the regulation of glucose utilization, lipid metabolism, and energy balance [5,6].

Myonectin (CTRP15), a member of the C1q/TNF-related protein family, has been identified as a nutrient-responsive myokine predominantly synthesized in skeletal muscle and released into the circulation [5]. Experimental studies have demonstrated that myonectin promotes the uptake and clearance of circulating free fatty acids by increasing the expression of fatty acid transport proteins such as CD36 and FATP1 in the liver and adipose tissue [5,6]. Circulating myonectin levels are known to respond dynamically to changes in nutritional status and physical activity. In animal models of obesity, myonectin deficiency has been linked to impaired lipid metabolism and increased susceptibility to hepatic steatosis and insulin resistance [7,8]. However, evidence regarding alterations in circulating myonectin levels in humans—particularly in pediatric obesity—remains limited and somewhat inconsistent [9,10].

Oxytocin is a hypothalamic neuropeptide traditionally known for its role in reproductive physiology, yet growing evidence indicates that it also participates in the regulation of appetite and energy expenditure [11]. The anorexigenic properties of oxytocin and its potential metabolic effects have attracted increasing attention in recent years. Nevertheless, studies examining circulating oxytocin levels in individuals with obesity have produced heterogeneous findings. While some investigations have reported reduced oxytocin concentrations, others suggest that obesity may be associated with compensatory changes in oxytocin signaling or resistance mechanisms analogous to leptin resistance [12,13]. In children and adolescents, the direction and clinical significance of these alterations remain unclear.

Simultaneous evaluation of muscle-derived myokines and neuroendocrine hormones involved in energy regulation may provide a more integrated understanding of the metabolic adaptations occurring in pediatric obesity. However, studies investigating the relationship between myonectin and oxytocin in this population are extremely limited. Therefore, the aim of the present study was to compare circulating myonectin and oxytocin levels between children with obesity and healthy controls and to explore their associations with insulin resistance and metabolic risk parameters.

## 2. Materials and Methods

### 2.1. Study Design and Participants

This cross-sectional, case–control study was conducted between January 2022 and December 2022 at the Pediatric Endocrinology Outpatient Clinic of Konya City Hospital. The study group consisted of 53 children with obesity aged 10.17–17.72 years, with a body mass index standard deviation score (BMI-SDS) ranging from 2.05 to 4.20. The control group included 37 healthy children of similar age and sex distribution without obesity, with BMI-SDS values between −1.89 and 1.53. The control group was composed of children who presented for evaluation of growth and development and were confirmed to be clinically and biochemically healthy.

Exclusion criteria were defined as syndromic obesity; known chronic systemic diseases (such as diabetes, hypothyroidism, Cushing syndrome, etc.); presence of acute infection; and a history of medication use within the past three months that could affect glucose or lipid metabolism. Pubertal staging was performed according to Tanner criteria by an experienced pediatric endocrinologist, and all participants were in the pubertal period.

The study protocol was approved by the Ethics Committee of Selçuk University Faculty of Medicine (Date: 9 November 2021, Decision No: 2021/492) and was conducted in accordance with the principles of the Declaration of Helsinki.

### 2.2. Anthropometric Measurements

Body weight and height of all participants were measured using calibrated standard devices, with participants wearing light clothing and no shoes. Height was measured to the nearest 0.1 cm using a Harpenden stadiometer (Holtain Ltd., Crymych, UK), and body weight was measured to the nearest 0.1 kg using a digital scale (Seca, Hamburg, Germany). BMI was calculated as weight divided by height squared (kg/m^2^). According to reference data for the Turkish pediatric population, children with a BMI above +2.0 SDS were classified as obese [14]. Weight-SDS, height-SDS, and BMI-SDS values adjusted for age and sex were calculated using national reference data developed for Turkish children through the “ÇEDD Çözüm/TPEDS Metrics” program [15].

### 2.3. Biochemical Analyses

Fasting venous blood samples were obtained in the morning (08:00–10:00). After centrifugation at 3000 rpm for 10 min, serum fractions were separated. Routine biochemical parameters were analyzed immediately, whereas aliquots intended for myonectin and oxytocin measurements were stored at −80 °C until analysis.

Fasting blood glucose (FBG), triglyceride, and high-density lipoprotein cholesterol (HDL-C) levels were measured using a colorimetric enzymatic method on an autoanalyzer (Roche Diagnostics, Mannheim, Germany). Serum insulin levels were determined by chemiluminescent immunoassay (Siemens Healthcare Diagnostics, IMMULITE 2000 XPi, Camberley, UK). Insulin resistance was assessed using the Homeostasis Model Assessment of Insulin Resistance (HOMA-IR) index, calculated as follows: Fasting Insulin (µU/mL) × Fasting Glucose (mg/dL)/405 [16].

### 2.4. Measurement of Serum Myonectin and Oxytocin

Circulating myonectin and oxytocin concentrations were determined using commercially available sandwich ELISA kits following the manufacturers’ instructions.

Serum myonectin levels were measured using a commercial ELISA kit from Bioassay Technology Laboratory (Shanghai, China; Catalog No: E5005Hu). The assay principle is based on a sandwich complex using antibodies specific to human myonectin. For this kit, the assay range was 0.05–10 ng/mL, and the minimum detectable concentration (sensitivity) was 0.03 ng/mL.

Serum oxytocin levels were determined using a specific ELISA kit from Bioassay Technology Laboratory (Shanghai, China; Catalog No: E1046Hu). The assay range of this kit was 2–600 pg/mL, with an analytical sensitivity of 1.06 pg/mL. Oxytocin measurements were performed according to the manufacturer’s protocol, and no solid-phase extraction procedure was applied to the samples prior to analysis. Samples with concentrations above the upper limit of the standard curve were diluted and reanalyzed according to the manufacturer’s instructions, and final concentrations were calculated after multiplying by the dilution factor.

In both assays, absorbance values were measured within 10 min after stopping the reaction at a wavelength of 450 nm using a microplate reader (Alisei, Radim Company, Rome, Italy). Sample concentrations were calculated using standard curves. The coefficients of variation (CV) indicating the reproducibility of the kits used in the study were reported as intra-assay <8% and inter-assay <10% for both myonectin and oxytocin. Washing procedures were standardized using an automatic plate washer (CombiWash, Human Diagnostics, Wiesbaden, Germany). All samples were analyzed in duplicate, and mean values were used for statistical evaluation.

### 2.5. Sample Size and Power Analysis

The sample size of the study was calculated using a priori power analysis with G*Power software (version 3.1.9.7; Franz Faul, Kiel University, Kiel, Germany). Based on data from similar studies investigating obesity and metabolic markers in the literature (Petro et al. [10] and Binay et al. [17]), the anticipated effect size (Cohen’s d) for detecting a significant difference in biomarker levels between obese and control groups was determined as 0.71. The type I error rate (α) was set at 0.05, and the target statistical power (1 − β) was set at 80%. Under these parameters and assuming a balanced group distribution, the minimum required total sample size to achieve statistical significance was calculated as 82 participants. In our study, this threshold was exceeded, and a total of 90 participants (53 obese, 37 controls) were included in the analysis, maintaining statistical power above 80%.

### 2.6. Statistical Analysis

Statistical analyses were conducted using IBM SPSS Statistics version 22.0 (IBM Corp., Armonk, NY, USA). Distribution of continuous variables was assessed using the Shapiro–Wilk test together with graphical inspection.

Variables with normal distribution are presented as mean ± standard deviation, whereas non-normally distributed variables are summarized as median with inter-quartile range (IQR: 25th–75th percentile). Group comparisons were performed using the independent samples *t*-test for normally distributed variables and the Mann–Whitney U test for non-normal variables. Categorical variables were compared using the chi-square test.

Since the raw data for serum myonectin and oxytocin levels did not show normal distribution, logarithmic transformation (log10) was applied to both parameters to stabilize variance. Log-transformed myonectin values demonstrated normal distribution; therefore, the independent samples *t*-test was used for group comparisons. Log-transformed oxytocin values did not show normal distribution; thus, the Mann–Whitney U test was preferred.

Associations between serum myonectin and oxytocin levels and age, BMI-SDS, and metabolic parameters (FBG, insulin, HOMA-IR, triglyceride, HDL-C) were evaluated using Spearman correlation analysis. Additionally, exploratory correlation analyses conducted within the obese subgroup were adjusted for multiple testing using Bonferroni correction to reduce the risk of type I error. To account for potential confounders, multivariable linear regression analyses were performed using two models (Model 1 and Model 2). Model 1 (demographic model) included obesity status, age, sex, BMI-SDS, and pubertal stage as independent variables. Model 2 (metabolic model) additionally incorporated metabolic parameters including HOMA-IR, triglycerides, and HDL-cholesterol.

Statistical tests were performed on log-transformed data; however, to facilitate clinical interpretation, results are presented in tables and text as raw values (mean ± SD for normally distributed variables and median [IQR] for non-normally distributed variables). A *p* value < 0.05 was considered statistically significant.

## 3. Results

### 3.1. Clinical and Metabolic Characteristics

The study group consisted of 53 children with obesity (mean age 14.29 ± 2.05 years) and 37 healthy controls (mean age 13.54 ± 1.87 years). There were no statistically significant differences between the groups in terms of age (*p* = 0.117), sex distribution (*p* = 0.491). However, the distribution of pubertal stages differed between groups (*p* < 0.001). Therefore, pubertal stage was included as a covariate in subsequent multivariable regression analyses to account for its potential confounding effect. Body weight, BMI, and BMI-SDS values were significantly higher in the obese group compared with the control group (*p* < 0.001).

Regarding metabolic parameters, fasting blood glucose (FBG) levels were similar between the groups (*p* = 0.146). However, fasting insulin and HOMA-IR levels were significantly higher in the obese group compared with controls (*p* < 0.001). In addition, the obese group had higher triglyceride levels (*p* = 0.011) and lower HDL-C levels (*p* = 0.026) than the control group (Table 1).

### 3.2. Serum Myonectin and Oxytocin Levels

**Figure 1:**
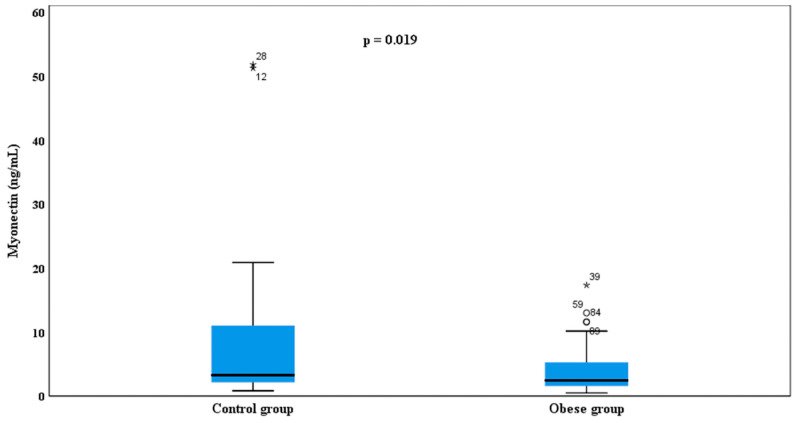
Comparison of serum myonectin levels between obese children and healthy controls. Data are presented as box-and-whisker plots based on raw values. Boxes represent the median (horizontal line) and the interquartile range (IQR; 25th–75th percentiles). Whiskers extend to 1.5 × IQR from the quartiles. Circles (∘) indicate outliers (>1.5 × IQR), and asterisks (*) indicate extreme outliers (>3 × IQR). Statistical significance (*p* < 0.05) was determined using the independent-samples *t*-test performed on log-transformed data.

Analyses performed on log-transformed data demonstrated that serum myonectin and oxytocin levels were influenced by the presence of obesity. Serum myonectin levels were significantly lower in children with obesity (mean 4.01 ± 3.66 ng/mL) compared with healthy controls (8.35 ± 12.00 ng/mL) (*p* = 0.019) (Figure 1).

**Figure 2:**
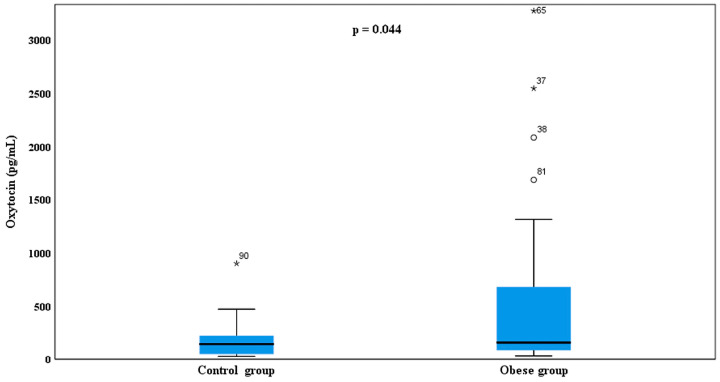
Comparison of serum oxytocin levels between obese children and healthy controls. Data are presented as box-and-whisker plots based on raw values. Boxes represent the median (horizontal line) and the interquartile range (IQR; 25th–75th percentiles). Whiskers extend to 1.5 × IQR from the quartiles. Circles (∘) indicate outliers (>1.5 × IQR), and asterisks (*) indicate extreme outliers (>3 × IQR). Statistical significance (*p* < 0.05) was assessed using the Mann–Whitney U test performed on log-transformed data.

In contrast, serum oxytocin levels were significantly higher in the obese group [median (IQR) 156.2 (83.9–754.9) pg/mL] compared with the control group [141.7 (47.7–221.5) pg/mL] (*p* = 0.044) (Figure 2).

### 3.3. Associations Between Biomarkers and Metabolic Parameters

Spearman correlation analyses performed within the obese group revealed no significant association between serum myonectin and oxytocin levels (*r* = −0.104, *p* = 0.468). Furthermore, neither hormone level showed a significant correlation with age, BMI-SDS, fasting insulin, HOMA-IR, triglyceride, or HDL-C (all *p* > 0.05) (Table 2). Exploratory analyses corrected for multiple testing using Bonferroni adjustment are provided in Table S1.

Multivariable linear regression analyses were performed to account for potential confounding factors (Table S2). In Model 1, adjusting for obesity status, age, sex, BMI-SDS, and pubertal stage, pubertal stage was independently associated with circulating myonectin levels (β = −0.124, *p* = 0.040). In Model 2, which additionally included metabolic parameters (HOMA-IR, triglycerides, and HDL-cholesterol), this association showed borderline significance (β = −0.121, *p* = 0.051). No independent associations were observed between metabolic parameters and circulating oxytocin levels.

## 4. Discussion

The present study demonstrates that circulating concentrations of the muscle-derived myokine myonectin and the neuropeptide oxytocin differ significantly between children with obesity and healthy controls. Specifically, children with obesity exhibited lower serum myonectin levels and higher oxytocin concentrations. These findings support the concept that pediatric obesity may involve alterations not only in adipose tissue biology but also in muscle-derived metabolic signaling and neuroendocrine regulation.

Reduced myonectin concentrations observed in the obesity group may indicate impaired skeletal muscle–mediated regulation of lipid metabolism. Myonectin has been reported to facilitate the uptake of circulating free fatty acids in metabolic tissues through the upregulation of fatty acid transport proteins [5,6]. In experimental models, myonectin deficiency has been associated with increased lipid storage [7], and inhibitory effects on adipogenesis have also been reported [8]. In our cohort, the coexistence of hypertriglyceridemia and lower circulating myonectin levels may suggest a disturbance in muscle-derived metabolic regulation in children with obesity. However, findings regarding the relationship between myokines and metabolic parameters in the pediatric population are not consistent. In a recent study investigating the association between hepatic steatosis and myokine levels in children, myonectin concentrations were reported to vary according to metabolic phenotype [18]. This variability suggests that alterations in myonectin may reflect a more complex and multifactorial metabolic adaptation rather than a unidirectional mechanism specific to obesity. Puberty is characterized by profound hormonal and metabolic changes, including transient physiological insulin resistance and alterations in anabolic hormone activity. During this period, increases in growth hormone, IGF-1, and sex steroids may influence skeletal muscle metabolism and endocrine function [19]. In our study, pubertal stage showed an independent association with circulating myonectin levels in the demographic model. Although this association was attenuated after additional adjustment for metabolic parameters, the direction of the relationship remained consistent. These findings suggest that pubertal maturation may influence circulating myonectin levels, potentially through metabolic changes occurring during adolescence. As a nutrient-sensing myokine, myonectin secretion is responsive to systemic metabolic status, including shifts in insulin sensitivity and body composition [20]. Therefore, the observed relationship may reflect physiological metabolic adaptations accompanying pubertal development rather than a direct causal mechanism. These observations highlight the importance of considering pubertal maturation when interpreting endocrine and metabolic biomarkers in pediatric populations.

The observation of higher oxytocin levels in children with obesity is also noteworthy. Oxytocin plays an important role in appetite regulation and energy balance [11,12,13]. Although limited studies in the pediatric population have reported alterations in oxytocin levels in children with obesity, the direction of these findings remains heterogeneous [17,21]. While some studies have reported lower oxytocin levels in obese children [21], others have demonstrated variable results associated with different metabolic profiles [17]. Therefore, elevated oxytocin levels in the context of obesity may reflect a potential compensatory response to increased metabolic load; however, this interpretation should be approached with caution. Moreover, the extent to which peripheral oxytocin levels reflect central effects remains uncertain, and further physiological studies are warranted. In addition, the demonstration that skeletal muscle is capable of synthesizing oxytocin [22] suggests that muscle–neuroendocrine interactions may be more complex than previously assumed. In addition, the relatively wide distribution and the presence of outliers in circulating oxytocin levels should be considered when interpreting these findings. Such variability has been reported in previous studies measuring peripheral oxytocin concentrations and may partly reflect both biological variability and methodological factors related to immunoassay-based measurements [23,24].

In our study, the absence of significant correlations between myonectin and oxytocin levels and HOMA-IR or BMI-SDS suggests that these biomarkers may be regulated independently of conventional metabolic parameters. In adult studies, variable associations between myonectin levels and insulin resistance have been reported [25,26]. In the pediatric population, it has similarly been observed that myokine levels do not always demonstrate linear relationships with metabolic parameters [27,28]. Given that myonectin is a dynamic hormone responsive to exercise and nutritional status [29,30], the lack of correlation in single-time-point measurements may be explained. A systematic review by Alzaabi et al. [31] emphasized that physical activity levels may influence the myonectin response and that sedentary lifestyle patterns in individuals with obesity could modulate this response. In this context, single fasting blood measurements may reflect short-term changes in physical activity or energy balance rather than chronic metabolic status. The lack of significant correlations between circulating hormones and metabolic parameters should be interpreted cautiously. The subgroup size of obese participants (n = 53) may limit the statistical power to detect modest associations. Therefore, the absence of correlations may partly reflect insufficient statistical power rather than true independence.

Alterations in myonectin levels have been reported in association with various metabolic disorders [32,33,34,35]. In particular, the demonstrated relationship between myonectin and metabolic dysfunction–associated steatotic liver disease and cardiometabolic risk markers [32] supports the notion that this myokine may be linked to systemic metabolic processes. Recent data investigating the relationship between hepatic steatosis and myokines in children [18] further support the concept that the muscle–liver axis may be affected early in pediatric obesity.

This study has several limitations. The cross-sectional design does not allow for the establishment of causal relationships. Additionally, dietary content and physical activity levels were not objectively assessed. Information on socioeconomic status and psychological stress was not systematically collected within the study protocol. As these factors may influence circulating myokines and neuroendocrine hormones, their potential confounding effects cannot be entirely excluded. In addition, the study population was recruited from a single tertiary care center, which may limit the generalizability of the findings to other pediatric populations with different ethnic, geographic, or socioeconomic backgrounds. Nevertheless, the study population represents a clinically well-characterized cohort of children with obesity evaluated in a tertiary pediatric endocrinology setting. Although pubertal stage differed between groups and may have influenced circulating hormone levels, multivariable regression analyses including pubertal stage as a covariate were performed to minimize potential confounding effects. Another methodological limitation relates to the measurement of oxytocin. The absence of an extraction step in serum oxytocin measurements may contribute to variability in immunoassay-based measurements. In addition, circulating oxytocin concentrations may be influenced by circadian rhythms and acute stress responses despite standardized sampling conditions. Previous studies have reported that immunoassay-based oxytocin measurements performed without extraction may show variability and reduced specificity [23,24]. Therefore, the oxytocin findings should be interpreted cautiously, particularly considering the known variability of peripheral oxytocin measurements.

## 5. Conclusions

In conclusion, the present study demonstrates that children with obesity exhibit lower circulating myonectin levels and higher oxytocin concentrations compared with healthy controls. These findings suggest that metabolic alterations associated with obesity may extend beyond adipose tissue expansion and may involve changes in both muscle-derived signaling pathways and neuroendocrine regulation. The lack of significant associations with conventional metabolic markers may indicate that these hormones reflect early metabolic adaptations rather than established metabolic dysfunction. Nevertheless, given the cross-sectional nature of the study, causal relationships cannot be determined. Prospective and mechanistic studies are required to clarify the role of myonectin and oxytocin in the pathophysiology of pediatric obesity.

## Figures and Tables

**Table 1:** Demographic, anthropometric, and biochemical characteristics of children with obesity and healthy controls.

Parameters	Obesity (n = 53)	Control (n = 37)	*p* Value	Statistical Test
Age (years)	14.29 ± 2.05	13.62 ± 1.87	*p* = 0.117	*t*-test
Sex n (%) (female/male)	38 (71.7)/15 (28.3)	24 (64.9)/13 (35.1)	*p* = 0.491	*χ* ^2^
Pubertal stage (Tanner), n (%) (II–III/IV–V)	5 (9.4)/48 (90.6)	15 (40.5)/22 (59.5)	*p* < 0.001	*χ* ^2^
Height (cm)	161.5 ± 9.2	155.8 ± 11.4	*p* = 0.010	*t*-test
Height SDS	0.30 ± 1.66	−0.21 ± 1.31	*p* = 0.118	*t*-test
Weight (kg)	84.1 (73.6–98.6)	49.2 (43.8–56.6)	*p* < 0.001	Mann–Whitney U
Weight SDS	3.03 ± 1.11	−0.22 ± 1.19	*p* < 0.001	*t*-test
BMI (kg/m^2^)	31.5 (29.5–35.3)	20.2 (18.6–21.9)	*p* < 0.001	Mann–Whitney U
BMI SDS	2.86 ± 0.55	−0.07 ± 0.96	*p* < 0.001	*t*-test
Fasting glucose (mg/dL)	92 (87–98)	91 (84–95)	*p* = 0.146	Mann–Whitney U
Fasting insulin (µU/mL)	27.0 (18.90–44.15)	10.65 (9.20–12.87)	*p* < 0.001	Mann–Whitney U
Triglycerides (mg/dL)	146.0 (89.5–177.0)	113.1 (88.5–146.5)	*p* = 0.011	Mann–Whitney U
HDL-C (mg/dL)	42.09 ± 8.70	45.97 ± 6.89	*p* = 0.026	*t*-test
HOMA-IR	6.56 (4.11–11.34)	2.37 (2.06–2.84)	*p* < 0.001	Mann–Whitney U
Myonectin (ng/mL) ^†^	4.01 ± 3.66	8.35 ± 12.00	*p* = 0.019	*t*-test
Oxytocin (pg/mL) ^†^	156.2 (83.9–754.9)	141.7 (47.7–221.5)	*p* = 0.044	Mann–Whitney U

Data are presented as mean ± standard deviation (SD) for normally distributed variables and as median (IQR: Q1–Q3) for non-normally distributed variables. Group comparisons were performed using the independent samples *t*-test for normally distributed variables and the Mann–Whitney U test for non-normally distributed variables. ^†^ For myonectin and oxytocin, statistical analyses were conducted using log-transformed values; raw (non-transformed) values are presented for clinical interpretability. Q1, 25th percentile; Q3, 75th percentile; *χ*^2^, chi-square test; SDS, standard deviation score; HDL-C, high-density lipoprotein cholesterol; HOMA-IR, homeostasis model assessment of insulin resistance. A *p* value < 0.05 was considered statistically significant.

**Table 2:** Correlations between oxytocin, myonectin, and metabolic parameters in obese group.

Parameters	Myonectin (ng/mL)		Oxytocin (pg/mL)	
	*r*	*p*	*r*	*p*
Age	−0.010	0.943	−0.094	0.513
BMI SDS	−0.116	0.407	0.117	0.412
Fasting glucose	−0.084	0.548	−0.153	0.283
Fasting insulin	0.095	0.499	−0.081	0.570
HOMA-IR	0.061	0.667	−0.076	0.598
Triglycerides	−0.109	0.437	−0.113	0.431
HDL-C	0.123	0.379	−0.059	0.678
Oxytocin	−0.104	0.468	-	-

Spearman correlation analysis was performed in the obese group. Log10-transformed values were used for oxytocin and myonectin, whereas raw values were used for other variables. BMI-SDS, body mass index standard deviation score. HOMA-IR, homeostasis model assessment for insulin resistance; HDL-C, high-density lipoprotein cholesterol. A *p* value < 0.05 was considered statistically significant.

## Data Availability

The data presented in this study are available on request from the corresponding author due to privacy and ethical restrictions involving pediatric participants.

## Supplementary Materials

The following supporting information can be downloaded at: https://www.mdpi.com/article/10.3390/children13030401/s1, Table S1. Exploratory Spearman correlations between circulating myonectin, oxytocin, and metabolic parameters in obese children; Table S2. Multivariable regression models for circulating myonectin and oxytocin levels.
