# Supplementary material for: Circulating Myonectin and Oxytocin Levels in Pediatric Obesity: A Comparative Study
Source: Children (Basel). 2026 Mar 13;13(3):401. doi: 10.3390/children13030401 (PMC13025749; doi:10.3390/children13030401)
Supplement: Supplementary file 1 [file children-13-00401-s001.zip › children-4187182-supplementary.pdf]

Table S1. Exploratory Spearman correlations between circulating myonectin, oxytocin, and metabolic parameters in obese children (n = 53).

Variable	Myonectin r	p	Oxytocin r	p
**Age**	-0.01	0.943	-0.09	0.513
**BMI-SDS**	-0.12	0.407	0.12	0.412
**Fasting glucose**	-0.08	0.548	-0.15	0.283
**Insulin**	0.10	0.498	-0.08	0.570
**HOMA-IR**	0.06	0.667	-0.08	0.598
**Triglycerides**	-0.11	0.437	-0.11	0.431
**HDL-C**	0.12	0.379	-0.06	0.678

Table note:
Spearman correlation analysis was performed in the obese subgroup. To account for multiple testing, Bonferroni correction was applied (adjusted significance threshold $p < 0.0036$). None of the correlations remained statistically significant after correction.

Table S2. Multivariable regression models for circulating myonectin and oxytocin levels

Variable	Model 1 β (Myonectin)	p	Model 2 β (Myonectin)	p	Model 1 β (Oxytocin)	p	Model 2 β (Oxytocin)	p
**Obesity status**	-0.044	0.510	-0.051	0.454	0.061	0.474	0.054	0.538
**Age**	-0.000	0.997	-0.008	0.779	-0.028	0.404	-0.024	0.516
**Sex**	-0.102	0.314	-0.086	0.405	-0.143	0.262	-0.135	0.304
**BMI-SDS**	0.017	0.783	0.022	0.738	0.019	0.813	0.012	0.879
**Pubertal stage**	-0.124	0.040	-0.121	0.051	0.076	0.311	0.069	0.377
**HOMA-IR**	—	—	0.011	0.321	—	—	0.009	0.530
**Triglycerides**	—	—	-0.001	0.433	—	—	-0.001	0.592
**HDL-C**	—	—	0.005	0.447	—	—	-0.003	0.689

Model 1 (Demographic model): adjusted for obesity status, age, sex, BMI-SDS, and pubertal stage.
Model 2 (Metabolic model): additionally adjusted for metabolic parameters including HOMA-IR, triglycerides, and HDL-cholesterol.
Hormone levels were analyzed using log10-transformed values.
β values represent unstandardized regression coefficients.
